# Carrageenans and Their Oligosaccharides from Red Seaweeds *Ahnfeltiopsis flabelliformis* and *Mastocarpus pacificus* (Phyllophoraceae) and Their Antiproliferative Activity

**DOI:** 10.3390/ijms24087657

**Published:** 2023-04-21

**Authors:** Anna O. Kravchenko, Ekaterina S. Menchinskaya, Vladimir V. Isakov, Valery P. Glazunov, Irina M. Yermak

**Affiliations:** G.B. Elyakov Pacific Institute of Bioorganic Chemistry, Far Eastern Branch, Russian Academy of Sciences, 100 Let Vladivostoku Prosp., 159, 690022 Vladivostok, Russia; ekaterinamenchinskaya@gmail.com (E.S.M.); imyer@mail.ru (I.M.Y.)

**Keywords:** carrageenan, oligosaccharides, structure, anticancer activity, mild acid hydrolysis, NMR spectroscopy, Phyllophoraceae

## Abstract

Comparative structural analysis of gelling polysaccharides from *A. flabelliformis* and *M. pacificus* belonging to Phyllophoraceae and the effect of their structural features and molecular weight on human colon cancer cell lines (HT-29, DLD-1, HCT-116) was carried out. According to chemical analysis, IR and NMR spectroscopies, *M. pacificus* produces kappa/iota-carrageenan with a predominance of kappa units and minor amounts of mu and/or nu units, while the polysaccharide from *A. flabelliformis* is iota/kappa-carrageenan (predominance of iota units) and contains negligible amounts of beta- and nu-carrageenans. Iota/kappa- (Afg-OS) and kappa/iota-oligosaccharides (Mp-OS) were obtained from the original polysaccharides through mild acid hydrolysis. The content of more sulfated iota units in Afg-OS (iota/kappa 7:1) was higher than in Mp-OS (1.0:1.8). The poly- and oligosaccharides up to 1 mg/mL did not show a cytotoxic effect on all tested cell lines. Polysaccharides showed an antiproliferative effect only at 1 mg/mL. Oligosaccharides had a more pronounced effect on HT-29 and HCT-116 cells than the original polymers, while HCT-116 cells were slightly more sensitive to their action. Kappa/iota-oligosaccharides exhibit a greater antiproliferative effect and more strongly decrease the number of colonies forming in HCT-116 cells. At the same time, iota/kappa-oligosaccharides inhibit cell migration more strongly. Kappa/iota-oligosaccharides induce apoptosis in the SubG0 and G2/M phases, while iota/kappa-oligosaccharides in the SubG0 phase.

## 1. Introduction

Red seaweeds of Gigartinales are the source of two types of economically important sulfated polysaccharides—agars and carrageenans. The polymer chain of agar and carrageenan is built up from galactose residues linked by alternating β-(1→4)- and α-(1→3)-glycosidic bonds, forming the disaccharide unit. β-Galactose always has a D configuration, while α-galactose has a D or L configuration, depending on which galactans are divided into carrageenans and agars, respectively. In addition, α-linked galactose can be partially or completely in the form of a 3,6-anhydro derivative. Hydroxyl groups are sulfated, methylated or substituted with D-xylose, glucuronic or pyruvic acid [1,2]. It leads to large structural diversity in these polysaccharides. In addition to 3,6-anhydrogalactose, carrageenans differ from each other in the number and location of sulfate esters. This makes it possible to divide them into “types” and assign their names, where idealized structures are denoted in Greek letters, for example, kappa-, iota-, mu-, nu- and lambda-carrageenans [3]. Kappa- and iota-carrageenans contain 3,6-anhydro-α-D-galactose, which has a ^1^C_4_ conformation. It allows for a helicoidal secondary structure, which is essential for the gel-forming properties. In contrast, nu- and lambda-carrageenans contain 4-linked α-D-galactose 2,6-disulfate, which has a ^4^C_1_ conformation. Therefore, these polysaccharides are non-gelling at low salt concentrations (KCl, CaCl_2_) [4,5]. Natural polysaccharides rarely have a regular structure. As a rule, they are built up from disaccharide units of several carrageenan types with a predominance of one or another type [6,7,8], or they are even more complex D/L-hybrid structures that contain agar and carrageenan units [9,10,11].

The polysaccharide structure, in turn, depends on various factors, such as the environment [12,13,14,15], the life cycle stage [6,16,17,18] and macrophyte species [19]. For example, the accumulation of a polysaccharide with a high 3,6-anhydrogalactose amount in *Eucheuma isiforme* was noted during periods of high illumination [14]. There were more kappa units in kappa/iota-carrageenan from the gametophyte *Mastocarpus stellatus*, growing in areas exposed to strong winds and waves, as well as a sharper change in water temperature throughout the year, compared with seaweeds growing in relatively closed bays with less seasonal fluctuations in water temperature [15]. The tetrasporophytes of algae from Gigartinaceae and Phyllophoraceae produced mainly lambda family polysaccharides, while the female gametophyte–kappa- and iota-carrageenans have minor amounts of mu- and nu-carrageenans [16]. With regard to macrophyte species, algae *Kappaphycus alvarezii*, *K. striatum* and *K. malesianus* (Solieriaceae) produced kappa-carrageenan, while *Eucheuma denticulatum,* from the same family, synthesized iota-carrageenan [19].

The Phyllophoraceae is one of the largest families in Gigartinales and has about 132 members [20]. Among them, algal species of the genera Gymnogongrus, Ahnfeltiopsis, Stenogramme, Mastocarpus and Coccotylus are actively studied as sources of polysaccharides of interest in structure [6,9,10,21,22,23,24]. Previously, we showed the dependence of the polysaccharide structure from *Ahnfeltiopsis flabelliformis* on the phase of the algae life cycle and evaluated the structural features of the polysaccharide from *Mastocarpus pacificus* [10,25,26]. Since different seaweed species within a family can produce various structures of polysaccharides, the focus in this work is on a comparative structural study of the polysaccharides from *A. flabelliformis* and *M. pacificus* belonging to the same family.

Polysaccharides of red algae are interesting in that they have not only stabilizing, thickening and emulsifying properties that lead to their use in the food and cosmetic industries [27,28] but also exhibit a wide range of biological activity: antiviral [29,30], anticancer [31,32,33], immunomodulatory [5,34], antioxidant [35,36] and anticoagulant [37,38]. In recent years, studies conducted on various models of tumor cells in vivo and in vitro showed that sulfated polysaccharides of red seaweeds can act as anticancer agents by reducing cancer cell proliferation, activating immune cells and influencing caspase activation and apoptosis in tumor cells, while the observed effect is determined by the structural features of polysaccharides and molecular weight [31,33,39,40,41]. For example, iota-carrageenan has been shown to inhibit proliferation and cause the death of MDA-MB-231 breast cancer cells as a result of the activation of extrinsic apoptosis, as indicated by caspase 8 activation [40]. It has been established that kappa- and lambda-carrageenans reduced the viability of HeLa cells by 50 and 70%, respectively, at a concentration of 1 mg/mL, while affecting different phases of the cell cycle [41]. Ariffin and co-authors [39] showed that kappa-carrageenan oligosaccharides were more effective against Caco-2 cells than the original polysaccharide, while in the case of iota-carrageenan, the polysaccharide had a more pronounced effect than oligosaccharides. 

When investigating the anticancer activity of polysaccharides, it is important not only to evaluate their antiproliferative and antimetastatic properties but also to study the influence of structural features and molecular weight of carrageenans on their anticancer effect, which requires the production of oligosaccharides. Thus, the purpose of this article is comparative structural analysis of the poly- and oligosaccharides from *A. flabelliformis* and *M. pacificus* belonging to Phyllophoraceae collected in the Sea of Japan, and an investigation of the influence of structural features and molecular weight of carrageenans on their anticancer properties.

## 2. Results

### 2.1. Extraction, Fractionation and Chemical Analysis of Polysaccharides

Polysaccharides were isolated from *Ahnfeltiopsis flabelliformis* (cystocarpic plants) and *Mastocarpus pacificus* (gametophytes) (Phyllophoraceae) and fractionated with KCl and CaCl_2_, as described in the Experimental section. As a result, a gelling polysaccharide from *A. flabelliformis* was obtained, designated as Afg. Attempts to fractionate the polysaccharide from *M. pacificus* were unsuccessful. It was concluded that this polysaccharide is represented exclusively by the gelling fraction designated as Mp. 

The yields and composition of polysaccharides are presented in Table 1. Mp yield was 1.5-times higher than Afg. According to chemical analysis, both polysaccharides were highly sulfated galactans with galactose and 3,6-anhydrogalactose as the main monosaccharides, while Afg was more sulfated than Mp. Glucose and xylose were also present in minor amounts (Table 1). Glucose is typically the structural unit of floridean starch, which is α-(1→4)-D-glucan with branches at C-6 [42]. Xylose can be either a structural unit of the neutral polysaccharide–xylan or a single monosaccharide residue replacing a hydroxyl group of the galactan [1,2]. According to the partial reductive hydrolysis, Afg and Mp were carrageenans because they contained only disaccharide units of →3)-β-D-Galp-(1→4)-α-D-AnGalp-(1→ (carrabiose), and no agarose disaccharide repeating units were detected. According to HPLC, Afg and Mp were quite heterogeneous in molecular weight, with the average molecular weight of Afg being 309 kDa, while Mp was 414 kDa (Table 1).

It can be seen from the molar ratio that both polysaccharides contained some α-galactose 6-sulfate in the 4-linked residue, which is part of the biosynthetic precursors of gelling types of carrageenan in the polymer chain. To confirm this fact, Mp was subjected to alkaline treatment with sodium hydroxide in the presence of sodium borohydride according to the method proposed by Rees [43]. The modified polysaccharide was labelled as Mpa. The main monosaccharides of the Mpa were galactose and 3,6-anhydrogalactose in a molar ratio of Gal:AnGal of 1.0:0.9. It indicated that α-galactose 6-sulfate in the native polysaccharide was transformed into 3,6-anhydro-α-D-galactose (Table 1).

### 2.2. Structural Analysis of Afg and Mp Polysaccharides

#### 2.2.1. IR and NMR Spectroscopy Analysis

The structures of the isolated Afg and Mp polysaccharides were studied via FT-IR and NMR spectroscopies and compared with the structures of the carrageenans from these seaweeds that were established in detail by us earlier [10,26]. Absorption bands in the IR spectra and chemical shifts in the NMR spectra were assigned via comparison to the signals of known carrageenan structures [44,45,46,47].

The IR spectra of Afg (Figure S1) and Mp (Figure S2) were identical in the set of absorption bands. An intense absorption band at 1230–1250 cm^–1^ indicated the presence of a significant number of sulfate groups (–S=O asymmetric vibration) [47]. There were absorption bands at 932 (C-O vibration of 3,6-anhydrogalactose) and 849 cm^−1^ (the secondary axial sulfate group at C-4 of the 3-linked β-D-galactose) in both IR spectra that were characteristic of kappa-carrageenan [48]. An absorption band at 805 cm^−1^ was assigned to the secondary axial sulfate group at C-2 of the 4-linked 3,6-anhydro-α-D-galactose of the iota-carrageenan [49]. It should be noted that the intensity of this absorption band relative to the absorption band at 849 cm^−1^ was much higher for Afg (Figure S1) than for Mp (Figure S2). This fact indicated the presence of a larger amount of iota-carrageenan in the first polysaccharide. The weak band at 890 cm^−1^ evinced the presence of unsulfated β-D-galactose, typical for beta-carrageenan [47].

Analysis of the NMR spectra of the polysaccharides confirmed the IR spectroscopy data. There were four signals with different intensities at 92.4, 95.5, 102.7 and 102.9 ppm in the anomeric area of the ^13^C NMR spectrum of the Mp (Table 2). Poorly resolved signals at 102.7 and 102.9 ppm were the result of the overlapping of C-1 signals of 3-linked β-D-galactose 4-sulfate of iota- (G4S’) and kappa-carrageenans (G4S), respectively. The signals at 92.4 and 95.5 ppm were assigned to C-1 of 4-linked 3,6-anhydro-α-D-galactose 2-sulfate (DA2S) and unsulfated 3,6-anhydro-α-D-galactose (DA) of iota- and kappa-carrageenans, respectively (Table 2). The ^13^C NMR spectrum in the upfield region was typical of kappa- and iota-carrageenans [44,45,46]. In addition, minor signals at 98.6 and 105.4 ppm were present in the anomeric region of the ^13^C NMR spectrum (not shown), which can be attributed to α-D-galactose 6-sulfate/2,6-disulfate and β-D-galactose 4-sulfate, respectively, of mu- and/or nu-carrageenans. 

As in the case of Mp, signals of different intensities at 92.9, 96.2, 102.9 and 103.1 ppm were present in the anomeric region of the ^13^C NMR spectrum of Afg. They corresponded to C-1 signals of 4-linked 3,6-anhydro-α-D-galactose 2-sulfate (DA2S), unsulfated 3,6-anhydro-α-D-galactose (DA) and 3-linked β-D-galactose 4-sulfate (G4S’, G4S) of iota- and kappa-carrageenans, respectively (Table 2). However, unlike Mp, there were two more signals at 95.9 and 103.2 ppm in the Afg NMR spectrum, which were assigned to C-1 of 4-linked 3,6-anhydro-α-D-galactose (DA’) and 3-linked β-D-galactose (G), respectively, of beta-carrageenan [44,45,46]. The ^13^C NMR spectrum in the upfield region was typical for iota-, kappa- and beta-carrageenans. In addition, the signal with a weak intensity at 5.50 ppm in the ^1^H NMR spectrum (not shown) was characteristic of the H-1 of α-D-galactose 2,6-disulfate of nu-carrageenan (the biosynthetic precursor of iota-carrageenan) [50]. According to NMR spectroscopy, the content of the iota-type disaccharide units in the Afg polymer chain was predominant, and the ratio of iota and kappa units was 2:1.

Thus, the polysaccharide from *M. pacificus* was kappa/iota-carrageenan with a predominance of kappa units and contained negligible amounts of biosynthetic precursors, while the gelling polysaccharide from *A. flabelliformis* was iota/kappa-carrageenan with a ratio iota/kappa of 2:1 and contained negligible amounts of beta- and nu-carrageenans.

#### 2.2.2. Mass Spectrometry Analysis of Mp and Af

To obtain information about minor components of the polysaccharides and to confirm their hybrid nature, mass spectrometry of oligosaccharides obtained by mild acid hydrolysis in heavy oxygen water (H_2_^18^O) for Mp and by enzymatic hydrolysis with iota-carrageenase for Af was used. The results of these studies were described in detail in our previous papers [10,26]. Therefore, only the most significant will be presented in this paper. It was shown that Mp mainly consisted of kappa di- and tetrasaccharide fragments. The hybrid nature of Mp was proved due to the presence of kappa/iota-tetrasaccharides in the oligosaccharide mixture. In addition, the presence of minor mu-carrageenan insertions (the precursor of kappa-carrageenan) in the polymer chain was established [26].

The combination of enzymatic hydrolysis, separation of the oligosaccharides by HPLC and ESI MS/MS made it possible to establish that the Af polysaccharide was built up mainly from tetrasaccharides of iota-carrageenan (27%) and hybrid hexasaccharides with various sequences of kappa and iota units (39%): (DA2S-G4S-DA2S-G4S-DA-G4S, DA-G4S-DA2S-G4S-DA2S-G4S, DA2S-G4S-DA-G4S-DA2S-G4S). Iota-carrabiose and hybrid kappa/iota-carrageenan tetrasaccharides were present as minor components [10].

### 2.3. Preparation and Characterization of Afg and Mp Oligosaccharides and Polysaccharides with a Lower Molecular Weight

It is known that the manifested biological effect is due not only to the structural features of polysaccharides but also to their molecular weight [39,51,52]. Therefore, to study the effect of the molecular weight of carrageenans on their anticancer activity, polysaccharides with a lower molecular weight were obtained from the Mp and Afg by mild acid hydrolysis due to an increase in the hydrolysis time (0.5, 1, 2, 4 and 6 h). The oligosaccharides were also obtained by hydrolysis of Mp and Afg for 24 and 44 h, respectively.

The analysis of the obtained polysaccharides with a lower molecular weight is presented in Table 3. As a result of hydrolysis, the sulfation degree of the samples differed slightly from each other and was comparable with the sulfation degree of the initial polymers (Table 1). According to ^1^H NMR, Afgh_1_-Afgh_5_, like the original Afg, were mainly iota/kappa-carrageenan with a predominance of iota units and contained minor amounts of nu-carrageenan, while Mph_1_-Mph_5_ were kappa/iota-carrageenan with a predominance of kappa units and included minor amounts of mu- and nu-carrageenans (Table 2). In addition, no significant structural changes occurred during hydrolysis in both cases. Since the differences in the molecular weights of Afgh_1_ and Afgh_2_, as well as Mph_1_ and Mph_2_, were insignificant, samples starting from Afgh_2_ and Mph_2_ were taken for further analysis.

Oligosaccharides obtained by mild acid hydrolysis from the original Afg and Mp polymers, designated as Afg-OS and Mp-OS, respectively, were characterized. According to chemical analysis, Afg-OS and Mp-OS, like Afg and Mp, were highly sulfated galactans with molecular weights of 9.1 and 2.4 kDa, respectively (Table 4). The yields of Afg-OS and Mp-OS amounted to more than half of the initial polysaccharides. It should be noted that 3,6-anhydrogalactose was slightly damaged during hydrolysis. It was a general trend for acid hydrolysis, observed in previously published works [53].

**Figure 1:**
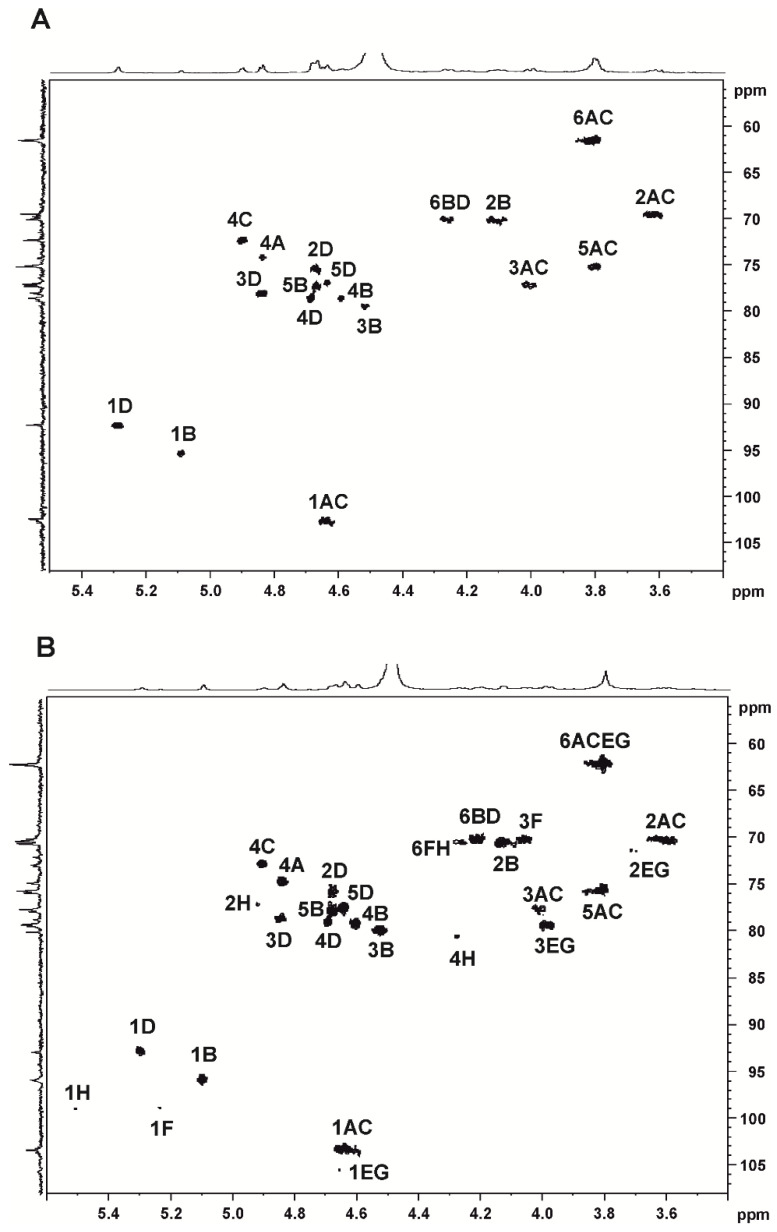
^13^C-^1^H HSQC NMR spectra of Afg-OS (**A**) and Mp-OS (**B**).

The structures of Afg-OS and Mp-OS were studied via 2D NMR spectroscopy (COSY and HSQC), which allowed us to assign the signals of monosaccharide residue protons and to carry out C/H correlation (Figure 1, Table S1). There were correlation signals of C-1/H-1 at 92.7/5.29, 95.6/5.09, 103.2/4.64 and 103.4/4.61 ppm, corresponding to four galactose residues in the anomeric area of HSQC spectra of both Afg-OS and Mp-OS (Figure 1, Table S1). The first two correlation signals corresponded to 4-linked 3,6-anhydro-α-D-galactose 2-sulfate (1D) and unsulfated 3,6-anhydro-α-D-galactose (1B), and the second two were characteristic of 3-linked β-D-galactose 4-sulfate of iota- (1C) and kappa-carrageenans (1A), respectively. These data were in good agreement with the NMR results of the original polysaccharides (Table 2). The identification of the remaining cross-peaks showed the presence of two disaccharide units differing from each other in the sulfate group at C-2 of 4-linked 3,6-anhydro-α-D-galactose. Therefore, the correlation signals at 75.5/4.67 and 70.4/4.13 ppm corresponded to C-2/H-2 sulfated (2D) and unsulfated (2B) 4-linked 3,6-anhydro-α-D-galactose, respectively. The presence of the sulfate group at C-2 of 4-linked 3,6-anhydro-α-D-galactose shifts the carbon signal by 5.1 ppm in the downfield compared to the carbon signal containing no substituent, which is an agreement with the literature data [45]. Two signals at 74.6/4.84 and 72.7/4.90 ppm were assigned to C-4/H-4 of 3-linked β-D-galactose 4-sulfate of kappa- (4A) and iota-carrageenans (4C), respectively. The integration of α-anomeric proton signals in ^1^H NMR spectra at 5.10 and 5.29 ppm made it possible to calculate the ratio of kappa and iota units in the samples under study. The iota/kappa ratio was 7:1 for Afg-OS, while it was 1.0:1.8 for Mp-OS. It should be noted that during hydrolysis, the number of kappa units significantly decreased in comparison with the initial polymers Afg and Mp.

In addition, there were correlation signals of C-1/H-1 at 98.6/5.26 and 98.9/5.50 ppm in the α-anomeric area of the HSQC spectrum of Mp-OS, which corresponded to 4-linked α-D-galactose 6-sulfate (1F) and α-D-galactose 2,6-sulfate (1H) of mu- and nu-carrageenans, respectively (Figure 1B, Table S1). The signal at 105.5/4.64 ppm was characteristic of 3-linked β-D-galactose 4-sulfate of mu- (1E) and nu-carrageenans (1G), respectively.

Thus, Afg-OS was iota/kappa-carrageenan with a predominance of iota units, while Mp-OS was kappa/iota-carrageenan with a predominance of kappa units and contained minor amounts of mu- and nu-carrageenans.

### 2.4. Cytotoxic and Antiproliferative Effect

The cytotoxic activity of poly- (Afg and Mp) and oligosaccharides (Afg-OS and Mp-OS) was evaluated towards such cell lines as human colon cancer cell lines HT-29, DLD-1, HCT-116 and human embryonic kidney HEK-293. The test compounds at concentrations up to 1 mg/mL did not show a cytotoxic effect on all tested cell lines when incubated for 24 h (Table 5). Polysaccharides Afg and Mp showed an antiproliferative effect only at a maximum concentration of 1 mg/mL. It should be noted that the highest percentage of viability inhibition was observed under the action of Afg, which contained more iota units, in relation to HT-29 cells, and it amounted to 34.7% of the control.

The hydrolyzed samples Afgh_2_-Afgh_5_ and Mph_2_-Mph_5_ did not have cytotoxicity and did not show any antiproliferative effect, so further studies were not carried out with them. 

**Figure 2:**
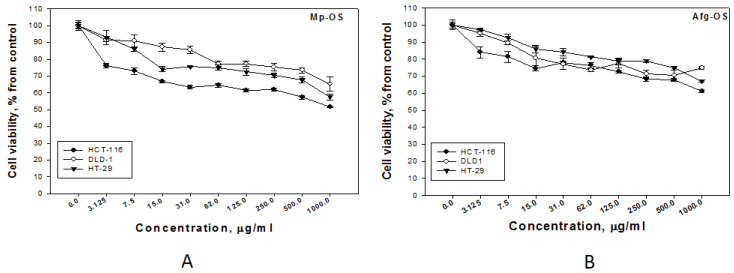
Antiproliferative effect of oligosaccharides Mp-OS (**A**) and Afg-OS (**B**) against three types of tumor cells (HCT-116, DLD-1, HT-29). Incubation time with cells—2 h.

Oligosaccharides Afg-OS and Mp-OS had a more pronounced effect on HT-29 and HCT-116 cells than the original polymers (Table 5). In addition, Mp-OS had a slightly more pronounced effect on DLD-1 cells compared to the polysaccharide. At the same time, Mp-OS had a greater cytotoxic and antiproliferative effect on DLD-1 and HCT-116 cells compared to Afg-OS. This fact may be due to the structural features of Mp-OS, which contains more kappa units (Figure 1). It should be noted that Afg-OS and Mp-OS at concentrations up to 125 μg/mL reduced the cell proliferation of HCT-116, HT-29 and DLD-1 by 30–35% compared to the control (Figure 2). A pronounced dose-dependent effect of oligosaccharides on colon adenocarcinoma cell lines was observed. The HCT-116 cell line was slightly more sensitive to the action of oligosaccharides, so these cells were chosen to further study the antitumor properties.

### 2.5. Oligosaccharides Inhibited HCT-116 Cell Migration

**Figure 3:**
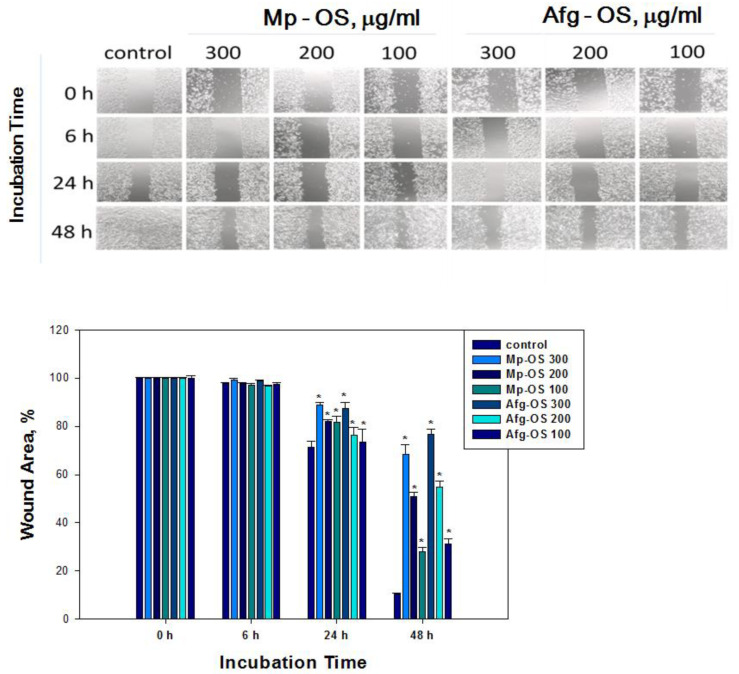
Cell migration into wound areas was observed under EVOS XL Core microscope at 0, 6, 24 and 48 h. ImageJ 1.52 software was used to analyze cell migration into wound areas. Data are presented as means ± SEM. * *p* value < 0.05 considered significant.

Cell migration is an important process in cancer metastasis; thus, the search for drugs that will help prevent this process remains relevant. The effects of oligosaccharides on cell migration were evaluated via an in vitro wound scratch migration assay. In the control group, HCT-116 cells migrated to completely close the wound areas at 48 h (Figure 3). However, in the treatment groups with oligosaccharides, cell migration into wound areas was significantly reduced at 6 h, 24 h and 48 h in both a dose- and time-dependent manner. Regardless of the concentration, the inhibitory effect of Afg-OS was slightly more pronounced than that of Mp-OS. The percentage of inhibition of cell migration for Mp-OS was 27.92 ± 1.69, 50.83 ± 1.78 and 68.51 ± 3.77% at 100, 200 and 300 μg/mL, respectively. The percentage of inhibition of cell migration for Afg-OS was 31.06 ± 2.26, 54.71 ± 2.62 and 76.63 ± 2.13% at 100, 200 and 300 μg/mL, respectively (Figure 3).

### 2.6. Oligosaccharides Reduced Number of Colonies Forming in HCT-116 Cells

The study of colony formation of tumor cells in vitro is based on the ability of 1 cell to turn into a colony, which should consist of at least 50 cells. Thus, the study of colony formation is a method that allows you to study each cell for the ability to grow indefinitely. In a living organism, this tendency for uncontrolled growth of tumor cells leads to tumor metastasis [55].

**Figure 4:**
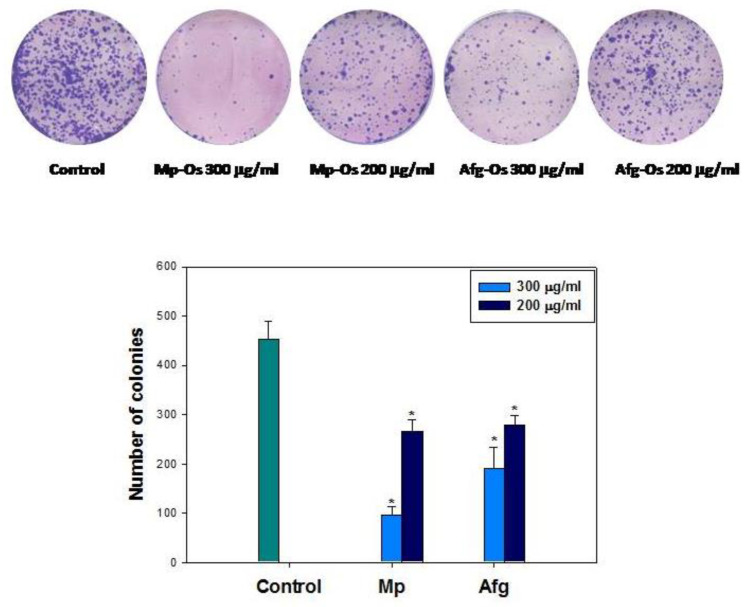
Colony images under different treatments. Oligosaccharides Mp-OS and Afg-OS reduced number of HCT-116 cell colonies in a dose-dependent manner. ImageJ 1.52 software was used to count the cell colonies. Data are presented as means ± SEM. * *p* value < 0.05 considered significant.

A study was conducted of the effect of oligosaccharides on the formation of colonies of HCT-116 tumor cells. The maximum inhibitory effect was observed for both oligosaccharides at a concentration of 300 μg/mL and amounted to 71.01 ± 8.37% for Mp-OS and 58.15 ± 2.16% for Afg-OS, respectively. It was found that the Mp-OS at a concentration of 200 μg/mL also significantly blocks the growth of tumor colonies. An inhibition of about 41.63 ± 4.55% of the control was observed (Figure 4). Afg-OS at a concentration of 200 μg/mL showed less of an effect on colony formation. Growth inhibition was approximately 38.55 ± 5.03% of the control.

### 2.7. Oligosaccharides Induced Apoptosis in HCT-116 Cells

**Figure 5:**
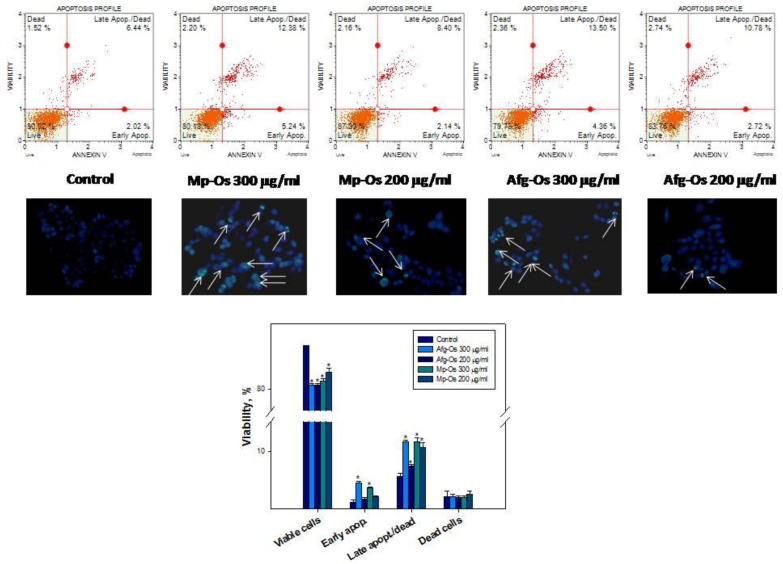
Hoechst 33342 staining showing chromatin condensation and DNA fragmentation in apoptotic cells under treatment of control, Mp-OS 300 and 200 mg/mL and Afg-OS 300 and 200 mg/mL. Arrows indicate nuclei with condensed chromatin. Apoptotic profile (Muse™ Annexin V and Dead Cell Assay) for HCT-116 cells, control, Mp-OS and Afg-OS. In all cases, the profiles were determined 72 h after incubation with substances. Each graph has 4 quadrant markers representing different cellular conditions: upper-left quadrant contains dead cells (dead cells/necrosis), upper-right quadrant contains late apoptotic/dead cells (cells positive for both annexin V and cell death marker 7-AAD), lower left contains live cells and lower right contains early apoptotic cells (cells positive for annexin V only). Data are presented as means ± SEM. * *p* value < 0.05 considered significant.

The Mp-OS and Afg-OS reduce proliferation and migration and also inhibit the growth of tumor cell colonies. Fluorescent dye Hoechst 33342 was used for staining to reveal morphological changes during apoptosis. The control group showed a normal nucleated cell composition; however, bright condensed blue fluorescence appeared in the cell nuclei in the treatment groups, indicating chromatin condensation and/or DNA fragmentation in oligosaccharide-treated cells (Figure 5).

When studying the induction of apoptosis through flow cytometry under the action of oligosaccharides, brightly fluorescent cells appeared in the dotogram in the lower-right quadrant, which are assessed as cells at the stage of early apoptosis. The appearance of cells in the right-upper quadrant indicates the binding of annexin V to phosphatidylserine (late apoptosis), as well as cell death (necrosis) by the penetration of 7-AAD into cells (Figure 5).

It was shown that the incubation of HCT-116 cells with the studied oligosaccharides leads to a significant decrease in living cells (lower-left quadrant). It was found that phosphatidylserine inversion on the outer monolayer of the plasma membrane of tumor cells of the HCT-116 line occurs under the action of the studied oligosaccharides for 72 h. Thus, the maximum effect was observed at a concentration of 300 μg/mL, both under the influence of Afg-OS and Mp-OS. The percentage of cells in early apoptosis, as well as in late apoptosis/necrosis, increased by 2- or more times compared to the control (Figure 5).

### 2.8. Oligosaccharides Induced Cell Cycle Arrest in HCT-116 Cells

**Figure 6:**
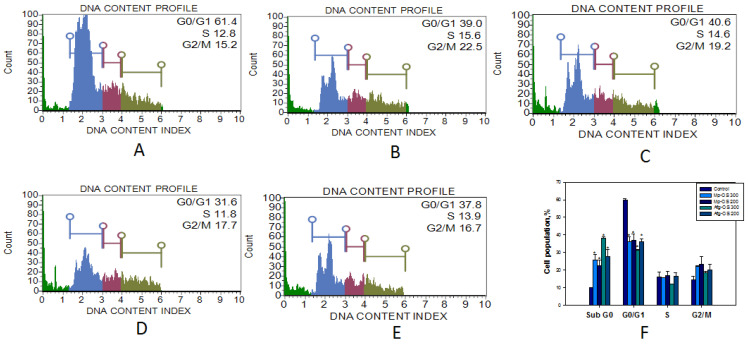
Histograms of the distribution of HCT-116 cells by phases in the control (**A**). Effect of Mp-OS ((**B**)-300 μg/mL, (**C**)-200 μg/mL) and Afg-OS (**D**)-300 μg/mL, (**E**)-200 μg/mL) on the distribution of HCT-116 cells by cell cycle phases. Graph of cell distribution by cell cycle phases (**F**). The time of incubation of oligosaccharides with cells is 72 h. Data are presented as means ± SEM. * *p* value < 0.05 considered significant.

It was found that the incubation of oligosaccharides Mp-OS and Afg-OS with HCT-116 cells causes a significant increase in the number of cells in the SubG0 phase, which corresponds to aneuploid cells and, consequently, the induction of apoptosis in these cells. Figure 6 shows histograms of the distribution of cells by cell cycle phases in the control and when exposed to Mp-OS and Afg-OS. The incubation of cells with Mp-OS resulted in an increase in aneuploid cells up to 25.9 ± 3.0% of the total number of cells. In addition, the Mp-OS increased the number of cells in the G2/M phase up to 22.1 ± 0.51%. Afg-OS was slightly more active in the SubG0 phase compared to Mp-OS. Thus, the histograms show that when HCT-116 cells are incubated, the number of cells in the SubG0 phase increases from 10 ± 0.1% in the control to 38.05 ± 0.85% and 27.55 ± 4.05% with the addition of Afg-OS at concentrations of 300 and 200 μg/mL, respectively. Figure 6A shows a histogram of control HCT-116 cells.

Thus, oligosaccharides had a more pronounced antiproliferative effect against human colon cancer cell lines than the original polysaccharides, while they showed the greatest sensitivity to HCT-116 cells. It has been shown that Mp-OS had a greater antiproliferative effect and more strongly reduced number of colonies forming in HCT-116 cells than Afg-OS, while Afg-OS inhibited cell migration more effectively. Both oligosaccharides induce apoptosis, with Mp-OS acting in both the SubG0 and G2/M phases and Afg-OS acting in the SubG0 phase.

## 3. Discussion

Due to the difficulties in identifying the chemical structure of algal sulfated polysaccharides, the relation between their structures and biological activities is not completely understood. The biological properties of carrageenans, including their antitumor potential, depend on their primary structure (structure of the basic disaccharide unit), number and location of the sulfate groups and degree of the molecular polymerization. Native carrageenans usually have a complex hybrid structure composed of different carrabiose types, the proportion and structure of which vary with algae species [19] and the life cycle stage [6,16,17,18]. 

In our work, polysaccharides isolated from *A. flabelliformis* and *M. pacificus* belonging to the same Phyllophoraceae family were used. It should be noted that both algae were collected from the Sea of Japan and were represented by gametophytes in the case of *M. pacificus* and cystocarpic plants in the case of *A. flabelliformis*. Gametophytes and cystocarpic plants of seaweeds of this family are known to produce hybrid carrageenans, consisting mainly of kappa and iota units with a predominance of one or another type of carrageenan, depending on the algae species, with minor inclusions of mu and/or nu units. At the same time, tetrasporophytes biosynthesize carrageenans of the lambda family, which mostly occur as non-cyclized structures, mainly lambda-carrageenan [56]. Like the polysaccharides from other members of the Phyllophoraceae studied by us, polysaccharides from *A. flabelliformis* and *M. pacificus* were sulfated galactans with minor amounts of glucose and xylose [9,57]. The yields of Afg and Mp were 21.2 and 31.5%, respectively, which are comparable with the polysaccharide yields from gametophytes and cystocarpic plants of other members of the family [21,22,57,58,59].

Previously, we studied [10,26], and in this work, it was confirmed that the polysaccharide from *M. pacificus* was kappa/iota-carrageenan with a predominance of kappa units and contained negligible amounts of biosynthetic precursors, while the gelling polysaccharide from *A. flabelliformis* was iota/kappa-carrageenan with a ratio iota/kappa of 2:1 and contained negligible amounts of beta- and nu-carrageenans. As for the *M. pacificus* studied by us, kappa/iota-carrageenan with a predominance of kappa units and minor amounts of mu- and nu-carrageenans was characteristic of gametophytes of *M. stellatus* [15,21]. On the contrary, for seaweeds of the Gymnogongrus (*G. torulosus*, *G. tenuis* and *G. griffithsiae*), as for the *A. flabelliformis* studied by us, carrageenans contained more iota than kappa units, and biosynthetic precursors were also present [9,22,57,59].

It is known that carrageenans are non-toxic compounds and have a variety of biological activities, including anticancer activity [31,32,33]. The biological effect manifested is influenced by both the structural features of the polysaccharide (the sulfation degree, the presence of 3,6-anhydrogalactose) and its molecular weight [31,33,39,40,41,60]. In this study, we used kappa/iota- (Mp) and iota/kappa- (Afg) carrageenans, both of which contain 3,6-anhydrogalactose and differ only in the ratio of kappa and iota units.

An analysis of the experimental data in vitro and in vivo indicates a high antitumor activity of both high-molecular-weight carrageenans and their low-molecular-weight derivatives [33,61,62]. Lower-molecular-weight carrageenans can be prepared by chemical or enzymatic depolymerization, while the antitumor activity of the derivatives may often be enhanced compared to the original polymers. Polysaccharides with a lower molecular weight and oligosaccharides were obtained to investigate the influence of the molecular weight of polysaccharides on the anticancer activity. We carried out mild acid hydrolysis of kappa/iota- and iota/kappa-carrageenans in 0.1 N HCl at 37 °C for 30 min, 1, 2, 4 and 6 h and obtained derivatives whose molecular weights were 2–16-times less than the initial polysaccharides. The low-molecular-weight derivatives (Afgh_2_-Afgh_5_ and Mph_2_-Mph_5_) did not have cytotoxicity and did not show antiproliferative effects. These samples did not show any significant effect, regardless of the molecular weight and structure.

To obtain oligosaccharides from Afg and Mp, different hydrolysis times were used. We found that 24 h was enough in the case of kappa/iota-carrageenan (Mp) and 44 h in the case of iota/kappa-carrageenan (Afg). The longer hydrolysis time for a polysaccharide with a large amount of iota units can be explained by the fact that 2-O-sulfation of 3,6-anhydrogalactose stabilizes the glycosidic bond at the reducing end and stronger conditions are required for its hydrolysis [63]. This statement was in good agreement with the results obtained, which showed that there were no significant changes in the ratio of iota and kappa units in the hydrolyzed samples Afgh_1_-Afgh_5_ and Mph_1_-Mph_5_ (from 0.5 to 6 h) in comparison with the initial polysaccharides. However, this ratio changed with longer hydrolysis time due to a decrease in kappa units, which are more sensitive to acid. This was especially noticeable for iota/kappa-carrageenan from *A. flabelliformis*, which was characterized by a predominance of iota units and underwent longer hydrolysis. At the same time, the content of more sulfated iota units in Afg-OS remains higher than in Mp-OS, as well as in native polysaccharides (iota/kappa 7:1 for Afg-OS and 1.0:1.8 for Mp-OS).

Colorectal cancer is quite common among both men and women throughout the world [64], and carrageenans, as dietary fibers, can be consumed orally; therefore, in this work, we investigated the anticancer effect of carrageenans against human colon cancer cells. Since polysaccharides can show different specificity of interaction to different cell lines, three cell lines, DLD-1, HT-29 and HCT-116, were taken.

Our poly- and oligosaccharides did not exhibit any cytotoxic effect at concentrations up to 1 mg/mL (incubation time–24 h), which is in good agreement with the action of other studied carrageenans [41,65] and, once again, confirms the non-toxicity of these natural substances. The studied polysaccharides had an antiproliferative effect only at a maximum concentration of 1 mg/mL, while iota/kappa-carrageenan proved to be the most effective against HT-29 cells and reduced cell viability by 34.7%. Literature data on the antiproliferative effect of various carrageenans are very ambiguous. This may be associated not only with the structural features of the polysaccharides themselves but also with the type of cancer cells, polymer concentration and treatment time. Thus, it was shown that kappa- and lambda-carrageenans at concentrations similar to ours exhibited high antiproliferative activity and reduced the viability of HeLa cells by 50 and 70%, respectively [41]. On the contrary, kappa- and iota-carrageenans at a concentration of 1 mg/mL (48 h incubation) reduced the viability of LM2 cells by 60%, while lambda-carrageenan only by 25% [61]. In addition, high antitumor activity was shown for kappa-carrageenan from *Kappaphycus alvarezii*: it inhibited the viability of MCF-7 cells by 55%, and HT-29, Hep G2 and MG63 by 65% at a concentration of 150 μg/mL (24 h incubation) [66]. In other studies, as in our work, the results indicate a low antiproliferative effect of initial carrageenans. For example, high-molecular-weight commercial kappa-carrageenan and kappa-carrageenan from *K. alvarezii* at 2 mg/mL (72 h incubation) reduced Caco-2 viability by 10 and 25%, respectively, and HepG2 viability by 0 and 15%, respectively, while high-molecular-weight commercial iota-carrageenan reduced viability by 25%, regardless of cell type [39]. Kappa-carrageenans (67 and 27 kDa) at a concentration of 1 mg/mL reduced the viability of HCT-116 cells (24 h incubation) by 16 and 27%, respectively [65].

In our case, oligosaccharides were more effective than the original polymers and reduced colon adenocarcinoma cell line proliferation by 30–35%, even at a concentration of 125 μg/mL. Since HCT-116 cells were more sensitive to oligosaccharides, further experiments were performed on this cell line. It should be noted that the less sulfated kappa/iota-oligosaccharide (Mp-OS) reduced the viability of HCT-116 cells at 1 mg/mL more than the iota/kappa-oligosaccharide (Afg-OS). It is believed that with an increase in sulfates, the interaction between carrageenan and cells may decrease, given that the membranes carry a negative charge [61]. The fact that oligosaccharides more strongly inhibit cancer cell proliferation than initial polysaccharides has been previously observed in studies on Caco-2 and HepG2 cells, where kappa-oligosaccharides derived from commercial kappa-carrageenan and kappa-carrageenan from *K. alvarezii* at a concentration of 2 mg/mL inhibited proliferation at 80–90%. As for iota-oligosaccharides, their effect was slightly higher than that of the original polymer but much less impressive than that of kappa-oligosaccharides [39]. An increase in the cytotoxicity of kappa- and lambda-oligosaccharides compared to polysaccharides was also shown on LM2 cells (48 h incubation) [61]. In addition, kappa-carrageenan oligosaccharides showed higher antitumor activity than polysaccharides against human nasopharyngeal carcinoma, gastric carcinoma and the HeLa cell line in vitro [67] and iota-carrageenan oligosaccharides against human osteosarcoma (HOS) in vitro and in vivo [68].

In the present study, we demonstrated the effect of sulfated oligosaccharides on cell cycle arrest, induction of apoptosis in the range of non-toxic concentrations and also showed their ability to significantly reduce the migratory ability in cells pretreated with the studied oligosaccharides compared with control cells. Oligosaccharides Mp-OS and Afg-OS arrested the cell cycle and delayed the cells in the G2/M phase in 72 h when incubated with HCT-116 cells. Considering the literature data, it was shown that carrageenans isolated from red algae block the cell cycle of tumor cells in different phases, as well as inducing apoptosis and suppressing migratory activity. Cell cycle arrest and initiation of apoptosis in cancer cells are the main events targeted by antitumor drugs. It was noted that commercial kappa-carrageenan, at a concentration of 250 μg/mL, increased the number of cells in the G2/M phase at 72 h in HeLa cells [41]. Other authors have shown that when LM2 cells are incubated with kappa-carrabiose at a concentration above IC50, the number of cells in the SubG0 phase increases, which corresponds to apoptotic cells, as well as increased arrest in the S phase (6.2 to 13.6%) and a corresponding decrease in cells in the G1 and G2/M phases as compared to the control. At the same time, non-toxic concentrations of kappa-carrabiose did not affect the phases of the cell cycle [61]. In the work of Raman M. and Doble M., it was found that exposure of human colon cancer cells, HCT116, to the sulfated fractions of kappa-carrageenan from marine red algae, *K. alvarezii* (F1 = MW 67 kDa and F2 = MW 27 kDa), resulted in apoptotic cell death and nuclear fragmentation [65]. Oligosaccharides of iota-carrageenan (native iota-carrageenan was purchased from Sigma) were observed to induce apoptosis and G1 phase arrest in human osteosarcoma cells [68].

In our work, Afg-OS, which contained more sulfated iota units, showed a slightly more pronounced effect on cell migration compared to Mp-OS. However, Calvo et al. reported that the strong migratory ability of untreated cells was dramatically impaired in cells pretreated with purified kappa-carrabiose over the entire concentration studied range [61].

Thus, oligosaccharides containing more sulfated iota units act more effectively on cell migration, while oligosaccharides with a high content of kappa units exhibit a greater antiproliferative effect and more strongly decrease the number of colonies forming in HCT-116 cells. Both oligosaccharides induce apoptosis, with Mp-OS acting in both the SubG0 and G2/M phases and Afg-OS acting in the SubG0 phase.

## 4. Materials and Methods

### 4.1. Algal Material

The alga *A. flabelliformis* was collected from Amur Bay (Sea of Japan) at 2 m depth in November 2015, while seaweed *M. pacificus* was harvested in Troitsa Bay (Sea of Japan) at 0.5 m depth in July 2016. Both seaweeds were washed with running water to clean epiphytes and remove soluble salts, and material was frozen in airproof plastic bags. The identification of algae and the determination of their morphological and anatomic characteristics were carried out using a light microscope by Dr. Oksana Belous from A.V. Zhirmunsky National Scientific Center of Marine Biology FEB RAS according to Perestenko [69]. *M. pacificus* was represented by gametophyte, whereas *A. flabelliformis* was represented by cystocarpic plants.

### 4.2. Isolation and Fractionation of Polysaccharides

Frozen algae (7–10 g) were crushed, added to distilled water at a ratio of 1:30 (*w*/*v*) and low-molecular-weight substances were extracted at a temperature of 20 °C for 12 h. The resulting extracts were filtered and discarded. The algal residues were re-suspended in distilled water at a ratio of 1:30 (*w*/*v*), and the polysaccharides were extracted three times at 80 °C for 3 h in a boiling water bath with constant stirring. Combined hot extracts were centrifuged at 12,000× *g*, 12 °C and for 30 min on Avanti JXN-30 centrifuge (Beckman Coulter, Brea, CA, USA) to remove cell wall residues, then filtered through a Vivaflow200 membrane (Sartorius, Göttingen, Germany) with a pore size of 100 kDa and concentrated on a rotary evaporator. The polysaccharides were precipitated with a triple volume of 96% ethanol. Then, the precipitates were centrifuged at 12,000× *g*, dissolved in distilled water, concentrated on a rotary evaporator and lyophilized. The polysaccharides from *A. flabelliformis* and *M. pacificus* were labelled as Af and Mp, respectively. The separation of polysaccharides into gelling and non-gelling fractions was performed as described in articles published by us earlier [10,26]. The Af gelled at a 4% CaCl_2_ fraction, designated as Afg, while Mp was completely represented by the gelling polysaccharides.

### 4.3. Structural Analysis 

#### 4.3.1. Analytical Methods 

Total reductive hydrolysis of the polysaccharides with 1 mL 2 M trifluoroacetic acid (TFA) (100 °C, 4 h) and 4-methyl-morpholine borane (40 mg) was carried out and then aldononitrile acetates were obtained to determine the content of 3,6-anhydrogylactose [70]. Other monosaccharides (galactose, glucose, xylose) were determined as alditol acetates [26]. The alditol and aldononitrile acetates of monosaccharides were analyzed by gas–liquid chromatography on a 6850 chromatograph (Agilent Technologies, Santa Clara, CA, USA) equipped with an HP-5MS capillary column (30 m × 0.25 mm, 5% Phenyl Methyl Siloxane) and a flame ionization detector. The analyses were carried out using a temperature gradient program from 150 to 230 °C; the rate of temperature change was 3 °C/min. The sulfate ester content in the polysaccharides was determined using the turbidimetric method [71]. The protein content in the polysaccharides was determined by Lowry’s method [72].

#### 4.3.2. Partial Reductive Hydrolysis

To determine the configuration of 4-linked 3,6-anhydrogalactose, partial reductive hydrolysis of Afg and Mp (5 mg) with 1 mL of 2 M TFA (65 °C, 8 h) and 4-methyl-morpholine borane (Sigma, USA) was carried out as described by us earlier [26]. Agarose (Sigma-Aldrich, St. Louis, MO, USA) and kappa-carrageenan from *Kappaphycus alvarezii* (Sigma-Aldrich, St. Louis, MO, USA) were used as standards for the production of aldononitrile acetates of agarobiose and carrabiose.

#### 4.3.3. Alkaline Treatment of Mp

The Mp (100 mg) was dissolved in 20 mL of distilled water, 20 mg of NaBH_4_ was added and solution was kept at 25 °C for 12 h. Then, 60 mg of NaBH_4_ and 800 mg of NaOH were added and mixture was heated at 80 °C for 6 h. After cooling, it was neutralized with acetic acid to pH 5.5, dialyzed against distilled water, concentrated on a rotary evaporator and lyophilized. The polysaccharide was labelled as Mpa.

#### 4.3.4. Fourier Transform–Infrared Spectroscopy (FT-IR) 

IR spectra of the studied polysaccharides were recorded in films on a Vector 22 Fourier transform spectrophotometer Equinox 55 (Bruker, Billerica, MA, USA) taking 120 scans with 4 cm^–1^ resolution. Compound (8 mg) was dissolved in H_2_O (1 mL) and heated at 37 °C on a polyethylene substrate until a dry film was produced. Then, the film was clamped between two NaCl plates, and the IR spectra were recorded in the 4000–600 cm^−1^ region. The spectra were cut out in the region of 1900–700 cm^−1^, and the baseline was corrected for scattering. The spectra were normalized by the absorption of the monosaccharide ring skeleton at ~1070 cm^−1^ (A_1070_ ≈ 1.0).

#### 4.3.5. NMR Spectroscopy 

Poly- (3 mg) and oligosaccharides (7 mg) were deuterium exchanged twice in D_2_O (0.6 mL) by freeze-drying prior to being examined in a solution of 99.95% D_2_O. ^1^H, ^13^C NMR, COSY and HSQC spectra of the samples were recorded with DRX-500 (125.75 MHz) spectrometer (Bruker, Bremen, MA, Germany) operating at 50 °C. Chemical shifts were described relative to acetone as internal standard (δ_C_ 31.45, δ_H_ 2.25). XWINNMR 1.2 software (Bruker, Bremen, MA, Germany) was used to acquire and process the NMR data.

#### 4.3.6. Preparation and Analysis of Oligosaccharides

##### Mild Acid Hydrolysis

To obtain oligosaccharides, Mp (5 mg) was dissolved in 1 mL of a 0.1 N HCl in H_2_^18^O and heated at 60 °C for 4 h. Hydrolysis was stopped by the addition of some drops of 0.5 M NH_4_OH to pH 8–9. The obtained oligosaccharides were stored at 4 °C and analyzed by mass spectrometry.

##### Enzymatic Hydrolysis

The enzymatic hydrolysis of Af was carried out with recombinant iota-carrageenase from *Microbulbifer thermotolerans*, as described by us earlier [10]. Iota-carrageenan from *Euchemia spinosum* (Sigma-Aldrich, St. Louis, MO, USA) was used as standard.

##### High-Performance Liquid Chromatography (HPLC)

Products of enzymatic hydrolysis of the Af were analyzed by HPLC using a Dionex Ultimate 3000 HPLC system, as described by Kravchenko and co-authors [26].

##### Mass Spectrometry

Matrix-assisted laser desorption/ionization mass spectra (MALDIMS) and electrospray ionization mass spectra (ESIMS) of obtained oligosaccharides were recorded with an Ultra Flex III MALDI-TOF/TOF mass spectrometer (Bruker, Bremen, MA, Germany) and with a Maxis Impact LC Q-TOF mass spectrometer (Bruker, Bremen, MA, Germany), respectively, in the modes described by us earlier [10,26].

### 4.4. Preparation of Afg and Mp Oligosaccharides and Polysaccharides with a Lower Molecular Weight

To reduce the molecular weight of polysaccharides, 5 portions of Afg and Mp samples (70 mg) were dissolved in 35 mL of 0.1 N HCl (2 mg/mL) and then hydrolyzed at 37 °C for 0.5, 1, 2, 4 and 6 h. The reaction was stopped by adding 0.5 M NH_4_HCO_3_ to pH 8–9. The neutralized hydrolysates were precipitated with 5 volumes of 96% ethanol, centrifuged at 8000× *g* for 30 min at 4 °C and the precipitates obtained were dissolved in water. The resulting samples were designated as Afgh_1_, Afgh_2_, Afgh_3_, Afgh_4_, Afgh_5_, Mph_1_, Mph_2_, Mph_3_, Mph_4_ and Mph_5_. Then, Afgh_1_, Afgh_2_, Mph_1_ and Mph_2_ were filtered through a Vivaflow200 membrane (Sartorius, Göttingen, Germany) with a pore size of 100 kDa, Afgh_3_, Afgh_4_, Mph_3_ and Mph_4_ through a Vivaflow50 membrane (Sartorius, Göttingen, Germany) with a pore size of 30 kDa and Afgh_5_ and Mph_5_ through a Vivaflow50 membrane with a pore size of 10 kDa. All samples were concentrated on a rotary evaporator and lyophilized.

To obtain oligosaccharides, the Mp and Afg (200 mg) were dissolved in 100 mL of 0.1 N HCl and heated at 37 °C for 24 and 44 h, respectively. Hydrolysis was stopped with the addition of some drops of 0.1 N NH_4_OH solution to pH 8–9. Then, oligosaccharides were precipitated with 5 volumes of 96% ethanol and centrifuged at 8000× *g* for 30 min at 4 °C. The precipitates were separated from supernatants, dialyzed against water (cut off 1 kDa) and lyophilized. The oligosaccharides were designated as Mp-OS and Afg-OS.

### 4.5. Molecular Weight Measurement

#### 4.5.1. Polysaccharides

Molecular weight distribution of the polysaccharides (5 mg/mL) was analyzed via HPLC using a chromatograph Agilent 1100 (Germany) equipped with a gel filtration column Shodex GS-620 and refractometer RID G1362A. The exclusion range was 10–1000 kDa. Filtered through a 0.1 micron membrane, 0.1 M LiNO_3_ was used as a mobile phase. Flow rate was 0.5 mL/min. Sample injection volume was 50 μL. The column was calibrated with sulfated dextrans with molecular weights 10, 40, 200 and 400–600 kDa (Sigma-Aldrich, St. Louis, USA).

#### 4.5.2. Oligosaccharides

The average molecular weight of Afg-OS and Mp-OS produced by mild acid hydrolysis was determined by the reducing sugars method with ferricyanide [54].

### 4.6. Biological Activity

#### 4.6.1. Cell Culture

Human HT29 (ATCC^®^HTB-38™), DLD-1 (ATCC^®^CCL-221™), HCT-116 (ATCC^®^CCL-247™) colon cancer cell lines and HEK-293 (ATCC^®^CRL-1573™) human embryonic kidney cell line were purchased from American Type Culture Collection (Manassas, VA, USA). The cells were cultured in a 75 cm^2^ flask containing RPMI-1640 media (Biolot, Saint Petersburg, Russia), 10% heat-inactivated FBS (Biolot, Saint Petersburg, Russia), 10,000 U/mL of penicillin and 10,000 μg/mL of streptomycin (Biolot, Saint Petersburg, Russia) and incubated at 37 °C with 5% CO_2_. After the cells reached confluence, the medium was removed, and cells were washed with phosphate-buffered saline (PBS) (Biolot, Saint Petersburg, Russia). Cells were then detached from culture flask by the addition of 0.25% Trypsin-EDTA solution (Biolot, Saint Petersburg, Russia) and were dispensed into new culture plates for further experiments.

#### 4.6.2. MTT Assay to Determine the Effect of Oligosaccharides on Cell Viability

The effect of oligosaccharides on the viability of human cancer and normal cells was determined via MTT (3-[4,5-dimethylthiazol-2-yl]-2,5 diphenyl tetrazolium bromide) assay. Briefly, 6 × 10^3^ cells per well were seeded into 96-well plates and incubated for 24 h for adhesion; then, they were incubated for 24 h and 72 h with different concentrations of oligosaccharides. After incubation, the medium with the test substances was replaced with 100 μL of fresh medium. Then, 10 μL of MTT (Sigma-Aldrich, St. Louis, USA) reagent (5 mg/mL MTT in PBS) was added into the cells and the plates for an additional 4 h. Thereafter, 100 μL of SDS-HCl solution (1 g SDS/10 mL dH_2_O/17 μL 6N HCl) was added to each well, followed by an 18 h incubation. The absorbance of the converted dye formazan was measured using a Multiskan FC microplate photometer (Thermo Scientific, Waltham, MA, USA) at a wavelength of 570 nm. All experiments were repeated in triplicate. Cytotoxic activity was expressed as a percentage of cell viability.

#### 4.6.3. Wound Scratch Migration Assay

To analyze the effect of test compounds on tumor cell migration, inserts from special migration plates (Culture-insert 2 Well 24, ibiTreat) were removed, leaving a gap of 500 ± 50 μm (according to the manufacturer’s data) between HCT-116 cells attached to the plate and the plastic bottom, which was, up to this point, separated by a silicone insert. Then, cells were washed twice with PBS to remove cell debris and floating cells before treatment with various concentrations of oligosaccharides for 0, 6, 24 and 48 h. Cells treated with culture medium only were used as vehicle control. Cell migration into the wound areas was then observed under an inverted microscope EVOS XL Core (Thermo Fisher Scientific, Waltham, MA, USA) with objective 10 x magnification.

#### 4.6.4. Colony Formation Assay to Determine the Effect on Cell Proliferation

The effect of oligosaccharides on the proliferation of HCT-116 cells was analyzed using the clonogenic assay [73]. Briefly, HCT-116 cells were cultured on 6-well plates at density of 1 × 10^3^ cells per well and received control media (RPMI-1640 media, 10% FBS, 10,000 U/mL of penicillin and 10,000 μg/mL of streptomycin) or media supplemented with 200 or 300 μg/mL of oligosaccharides. Cells were incubated for one week at 37 °C with 5% CO_2_ until the cells in control plates formed colonies that were visible to the eye and were of a substantially good size (at least 50 cells per colony). For fixation and staining, media were removed, and the cells were washed twice with PBS. The colonies were fixed with 0.5% crystal violet solution for 45 min at room temperature. The plates were then washed with water and air-dried.

#### 4.6.5. Annexin V Assay

HCT-116 cells were collected after incubation with oligosaccharides; then, cells were washed twice with cold PBS and incubated with trypsin-EDTA for detachment. The cell suspension was washed twice with centrifugation with PBS and 100 mL of Muse^®^ Annexin V, and Dead Cell reagent was added to each tube in accordance with manufacturer’s instructions (Luminex, Austin, TX, USA). The tubes were incubated for 20 min at RT. Then, fluorescence was measured with a Muse^®^ cell analyzer (Luminex, Austin, TX, USA), and data were processed by Muse 1.5 analysis software (Luminex, Austin, TX, USA). The proportion of apoptotic cells was expressed as a percentage.

#### 4.6.6. Hoechst 33342 Staining

HCT-116 cells were cultured on cover slides for 24 h and then treated with oligosaccharides. Cells treated with culture medium only were used as vehicle control. The cells were washed twice with PBS and fixed with 4% formaldehyde for 15 min at RT. After washing twice with PBS, the cells were permeabilized with 0.15% Triton-X 100 for 15 min at room temperature, followed by washing twice with PBS. An aliquot of 4 μg/mL of Hoechst 33342 solution was used to stain cells for 10 min in the dark at room temperature. Cover slides were then mounted on the slides, and DNA-fragmented or chromatin-condensed cells were observed under a fluorescence microscope (MIB-2-FL, LOMO, Russia) with objective 40× magnification [74].

#### 4.6.7. Cell Cycle Analysis

HCT-116 cells were treated with different oligosaccharide concentrations for 72 h. Cells treated with culture medium only were used as vehicle control. Cells were harvested, washed with PBS and fixed with ice-cold 70% ethanol in a dropwise manner prior to storage at −20 °C overnight. The cells were then washed with PBS, incubated with 200 μg/mL RNAse (PanReac, AppliChem, Darmstadt, Germany) and 20 μg/mL of propidium iodide (Sigma-Aldrich, St. Louis, MO, USA) for 30 min at 37 °C and the DNA content was analyzed with a Muse^®^ cell analyzer (Luminex, Austin, TX, USA). The data were processed using Muse 1.5 analysis software (Luminex, Austin, TX, USA). The proportion of cells in each phase of the cell cycle was expressed as a percentage.

## 5. Conclusions

Polysaccharides from red seaweeds *M. pacificus* and *A. flabelliformis* (Phyllophoraceae) were isolated and characterized via chemical analysis, IR and NMR spectroscopies. It was shown that the gelling polysaccharide from *A. flabelliformis* was hybrid iota/kappa-carrageenan, with an iota/kappa ratio of 2:1 and contained negligible amounts of beta- and nu-carrageenans. The polysaccharide from *M. pacificus* was kappa/iota-carrageenan with a predominance of kappa units and minor amounts of mu- and/or nu-carrageenans. Since the biological properties exhibited by carrageenans depend not only on their structural features but also on molecular weight, iota/kappa- and kappa/iota-oligosaccharides with molecular weights of 9.1 and 2.4 kDa, respectively, were obtained from the original polysaccharides through mild acid hydrolysis and characterized by chemical analysis and NMR spectroscopy. It was shown that iota/kappa-oligosaccharides had an iota/kappa unit ratio 7:1 and kappa/iota-oligosaccharides had a ratio of 1:1.8. The anticancer effect of carrageenans with different ratios of iota and kappa units and their oligosaccharides was studied on human colon cancer cell lines: HT-29, DLD-1 and HCT-116. We demonstrated that the poly- and oligosaccharides at concentrations up to 1 mg/mL were non-toxic to all tested cell lines. Polysaccharides showed an antiproliferative effect only at a maximum concentration of 1 mg/mL. Oligosaccharides had a more pronounced effect on HT-29 and HCT-116 cells than the original polymers and reduced the cell viability by 30–35% at a concentration of 125 μg/mL. The effect of sulfated oligosaccharides on cell cycle arrest, induction of apoptosis in the range of non-toxic concentrations and also their ability to inhibit the migratory ability in HCT-116 cells was shown. It should be noted that kappa/iota-oligosaccharides with a high content of kappa units exhibited a greater antiproliferative effect and more strongly decreased the number of colonies forming in HCT-116 cells. At the same time, iota/kappa-oligosaccharides containing more sulfated iota units inhibited cell migration more strongly. Kappa/iota-oligosaccharides induce apoptosis in the SubG0 and G2/M phases while iota/kappa-oligosaccharides in the SubG0 phase.

## Figures and Tables

**Table 1:** Yields and chemical analysis of polysaccharides (PS) from *A. flabelliformis* and *M. pacificus*.

Sample	PS Yield, % of Dried Algal Weight	Molecular Weight, kDa	Protein Content, %	Content, % of PS Sample Weight					Molar Ratio Gal:AnGal:SO_3_Na
				Gal	AnGal	Xyl	Glc	SO_3_Na	
Afg	21.2	309	7.0	29.2	18.1	1.5	0.0	33.8	1.0:0.7:1.8
Mp	31.5	414	2.7	36.1	22.8	0.8	0.2	26.3	1.0:0.7:1.1
Mpa	-	-	-	32.1	24.9	1.2	0.8	27.4	1.0:0.9:1.3

Note: Afg—gelling polysaccharides from *A. flabelliformis*; Mp—polysaccharides from *M. pacificus*; Mpa—Mp polysaccharides after 1 M NaOH treatment.

**Table 2:** ^1^H and ^13^C NMR signals (ppm) of Afg and Mp polysaccharides.

Polysaccharide	Carrageenan Type	MS Residue *	^13^C/^1^H Chemical Shifts (ppm)					
			C-1/H-1	C-2/H-2	C-3/H-3	C-4/H-4	C-5/H-5	C-6/H-6
Mp	kappa	G4S	102.9/4.64	70.0/3.60	79.2/3.99	74.4/4.84	75.3/3.80	61.8/3.80–3.70
		DA	95.5/5.09	70.0/4.05	79.6/4.52		77.5/4.60	70.2/4.20–3.62
	iota	G4S’	102.7/4.64	70.0/3.60	77.5/3.99	72.6/4.89	75.3/3.80	61.8/3.80–3.70
		DA2S	92.4/5.30	78.9/4.67	79.1/4.84		77.5/4.67	70.2/4.12–4.27
Afg	kappa	G4S	103.1/4.64	70.0/3.60	79.0/3.99	74.3/4.87	75.8/3.80	62.3/3.80–3.70
		DA	96.2/5.09	70.3/4.14	79.7/4.52	78.7/4.62	77.3/4.66	70.1/4.20–3.62
	iota	G4S’	102.9/4.64	70.3/3.60	77.8/3.99	72.9/4.89	75.8/3.79	62.3/3.80–3.70
		DA2S	92.9/5.30	77.9/4.65	78.8/4.82	77.9/4.76	76.9/4.75	70.1/4.12–4.27
	beta	G	103.2/4.61	70.1/3.62	80.6/3.86	66.7/4.14	76.1/3.70	62.3/3.80–3.70
		DA’	95.9/5.07	70.8/4.08	79.3/4.53	78.7/4.62	77.3/4.66	70.1/4.20–3.62

Note: MS—monosaccharide, *—nomenclature according to [3].

**Table 3:** Characterization of polysaccharides (PSs) with a lower molecular weight obtained by mild acid hydrolysis of Mp and Afg (0.1 N HCl, 37 °C).

Hydrolysis Time, h	Sample Name	SO_3_Na Content, % of PS Sample Weight	Molecular Weight *, kDa	^1^H NMR Data, Disaccharide Units Ratio
0.5	Afgh_1_	32.0	184	ι (5.30 ppm) (+); κ (5.10 ppm) (+); ν (5.50 ppm) (−) ι:κ = 2.4:1.0
1.0	Afgh_2_	33.9	160	ι (5.30 ppm) (+); κ (5.10 ppm) (+); ν (5.50 ppm) (−) ι:κ = 2.3:1.0
2.0	Afgh_3_	33.7	112	ι (5.30 ppm) (+); κ (5.10 ppm) (+); ν (5.50 ppm) (−) ι:κ = 2.8:1.0
4.0	Afgh_4_	31.2	56	ι (5.30 ppm) (+); κ (5.10 ppm) (+); ν (5.50 ppm) (−) ι:κ = 2.5:1.0
6.0	Afgh_5_	31.9	22	ι (5.30 ppm) (+); κ (5.10 ppm) (+); ν (5.50 ppm) (−) ι:κ = 2.5:1.0
0.5	Mph_1_	25.9	219	κ (5.10 ppm) (+); ι (5.30 ppm) (+); μ (5.26 ppm) (−); ν (5.30 ppm) (−); ι:κ = 1.0:3.0
1.0	Mph_2_	25.3	216	κ (5.10 ppm) (+); ι (5.30 ppm) (+); μ (5.26 ppm) (−); ν (5.30 ppm) (−); ι:κ = 1.0:2.9
2.0	Mph_3_	23.2	150	κ (5.10 ppm) (+); ι (5.30 ppm) (+); μ (5.26 ppm) (−); ν (5.30 ppm) (−); ι:κ = 1.0:3.1
4.0	Mph_4_	26.4	81	κ (5.10 ppm) (+); ι (5.30 ppm) (+); μ (5.26 ppm) (−); ν (5.30 ppm) (−); ι:κ = 1.0:3.1
6.0	Mph_5_	25.9	25	κ (5.10 ppm) (+); ι (5.30 ppm) (+); μ (5.26 ppm) (−); ν (5.30 ppm) (−); ι:κ = 1.0:3.2

Note: *—molecular weight measured by HPLC, (+)—major units, (−)—minor units.

**Table 4:** Yields and chemical analysis of oligosaccharides (OSs) from Afg and Mp.

Sample	Yield, % of PS Sample Weight	Molecular Weight *, kDa	Content, % of PS Sample Weight					Molar Ratio Gal:AnGal:SO_3_Na
			Gal	AnGal	Glc	Xyl	SO_3_Na	
Afg-OS	62.8	9.1	35.8	13.9	0.9	1.9	26.4	1.0:0.4:1.2
Mp-OS	73.5	2.4	38.7	19.2	1.1	0.0	27.4	1.0:0.6:1.1

Note: *—molecular weight measured according to [54].

**Table 5:** Cytotoxic and antiproliferative activity of the poly- and oligosaccharides from *A. flabelliformis* and *M. pacificus* against various types of tumor and normal human cells.

Compounds	DLD-1		HT-29		HCT-116		HEK-293	
	24 h	72 h	24 h	72 h	24 h	72 h	24 h	72 h
Afg-OS	-	25.3	-	43.4	-	38.2	-	41.6
Mp-OS	-	34.3	-	32.2	-	48.4	-	49.8
Afg	-	25.2	-	34.7	-	21.5	-	41.1
Mp	-	21.2	-	5.4	-	25.7	-	35.3

Note: The % inhibition of cell viability is shown at the maximum test concentration of 1 mg/mL when the cells were incubated for 72 h.

## Data Availability

Not applicable.

## Supplementary Materials

The following supporting information can be downloaded at: https://www.mdpi.com/article/10.3390/ijms24087657/s1.
