# Supplementary material for: Carrageenans and Their Oligosaccharides from Red Seaweeds Ahnfeltiopsis flabelliformis and Mastocarpus pacificus (Phyllophoraceae) and Their Antiproliferative Activity
Source: Int J Mol Sci. 2023 Apr 21;24(8):7657. doi: 10.3390/ijms24087657 (PMC10146057; doi:10.3390/ijms24087657)
Supplement: Supplementary file 1 [file ijms-24-07657-s001.zip › ijms-2276654-supplementary.pdf]

Supplementary Materials

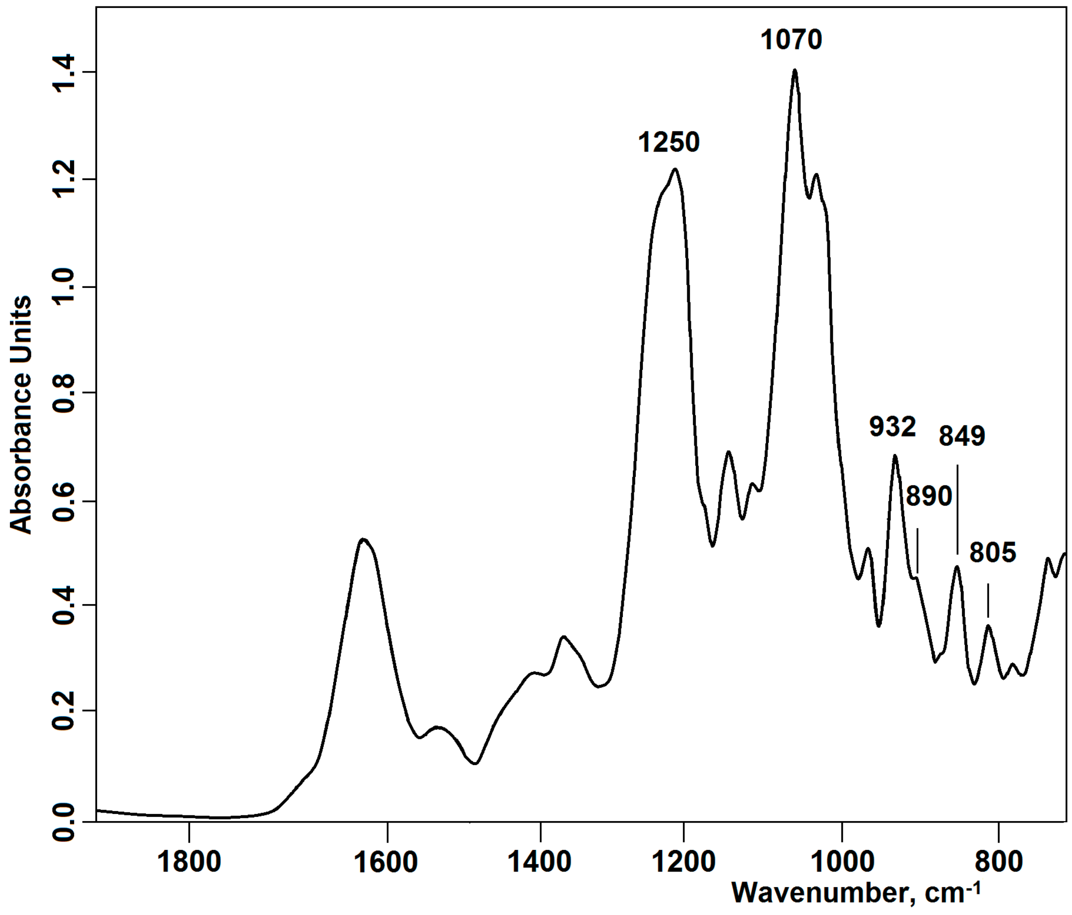

Figure S1. IR-spectrum of Afg

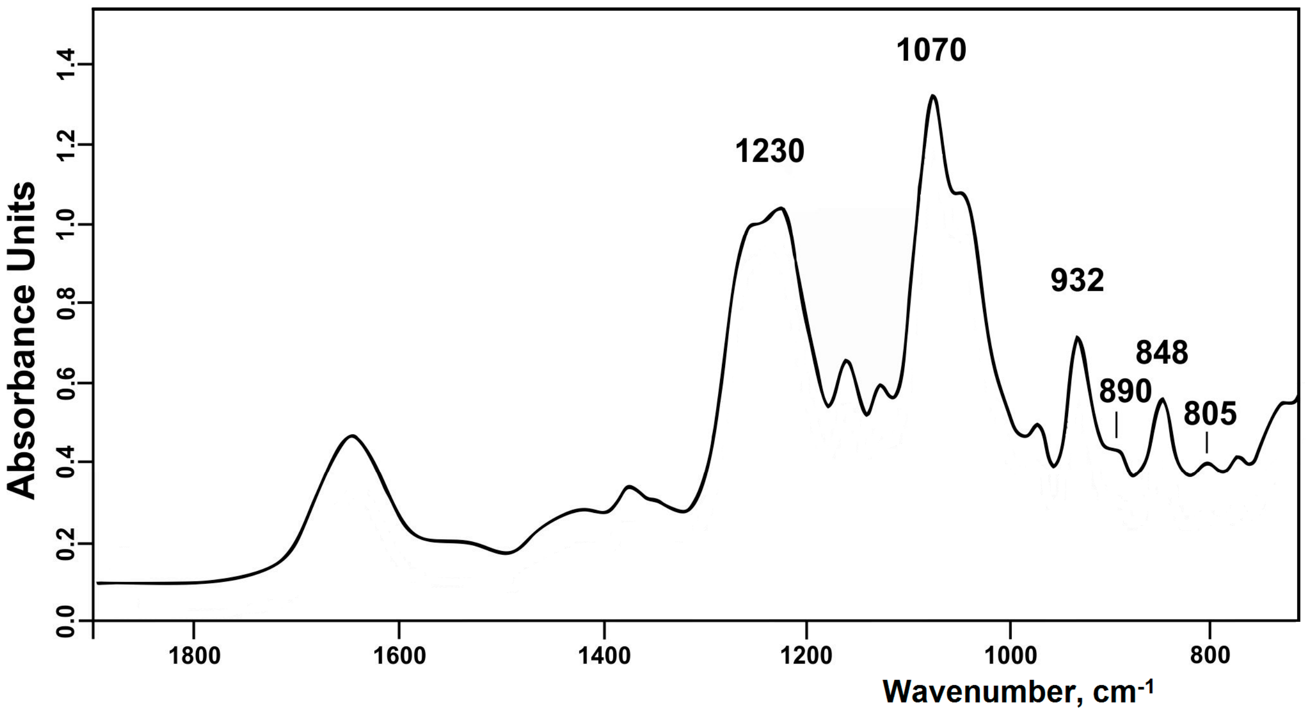

Figure S2. IR-spectrum of Mp

Table S1. ^{1}H and ^{13}C NMR signals (ppm) of Afg-OS and Mp-OS

Sample	Carrageenan type	MS residue	$^{13}C/^{1}H$ chemical shifts (ppm)					
			C-1/H-1	C-2/H-2	C-3/H-3	C-4/H-4	C-5/H-5	C-6/H-6
Afg-OS	kappa	A	102.7/4.61	69.7/3.61	77.4/4.00	74.4/4.84	75.6/3.79	62.0/3.80-3.70
		B	95.6/5.09	70.4/4.11	79.8/4.50	78.9/4.57	77.6/4.66	70.5/4.12-4.25
	iota	C	102.5/4.64	69.7/3.61	77.4/4.03	72.7/4.89	75.6/3.79	62.0/3.80-3.70
		D	92.7/5.29	75.7/4.66	78.4/4.83	78.9/4.67	77.3/4.62	70.5/4.12-4.25
Mp-OS	kappa	A	103.4/4.61	70.4/3.59	77.4/4.00	74.6/4.84	76.2/3.80	61.8/3.80-3.70
		B	95.6 /5.10	70.4/4.13	79.7/4.51	78.9/4.60	77.6/4.68	69.9/4.21
	iota	C	103.2/4.64	70.4/3.59	77.4/4.03	72.7/4.90	76.2/3.80	61.8/3.80-3.70
		D	92.5/5.29	75.5/4.67	78.5/4.84	79.0/4.69	77.2/4.63	70.1/4.21
	mu	E	105.5/4.64	71.1/3.70	79.2/3.97		75.4/3.81	62.0/3.80-3.70
		F	98.6/5.26		70.7/4.05		68.7/4.19	70.3/4.26
	nu	G	105.5/4.64	71.1/3.70	79.2/3.97		75.4/3.81	62.0/3.80-3.70
		H	98.9/5.50	77.0/4.92		80.9/4.29	68.7/4.19	70.3/4.26

Note: MS – monosaccharide; A – 3-linked β-D-galactose 4-sulfate, B – 4-linked 3,6-anhydro-α-D-galactose of kappa-carrageenan, C – 3-linked β-D-galactose 4-sulfate, D – 4-linked 3,6-anhydro-α-D-galactose 2-sulfate of iota-carrageenan, E – 3-linked β-D-galactose 4-sulfate, F – 4-linked α-D-galactose 6-sulfate of mu-carrageenan, G – 3-linked β-D-galactose 4-sulfate, H – 4-linked α-D-galactose 2,6-disulfate of nu-carrageenan
